# Are multidisciplinary teams in secondary care cost-effective? A systematic review of the literature

**DOI:** 10.1186/1478-7547-11-7

**Published:** 2013-04-04

**Authors:** K Melissa Ke, Jane M Blazeby, Sean Strong, Fran E Carroll, Andy R Ness, William Hollingworth

**Affiliations:** 1 School of Oral and Dental Sciences, University of Bristol, Lower Maudlin Street, Bristol BS1 2LY, UK; 2 School of Social and Community Medicine, University of Bristol, Canynge Hall, 39 Whatley Road, Bristol, BS8 2PS, UK; 3 University Hospitals Bristol NHS Foundation Trust, Dolphin House, Bristol Royal Infirmary, Bristol, BS2 8HW, UK

**Keywords:** Multidisciplinary teams, Cost-effective, Secondary care

## Abstract

**Objective:**

To investigate the cost effectiveness of management of patients within the context of a multidisciplinary team (MDT) meeting in cancer and non-cancer teams in secondary care.

**Design:**

Systematic review.

**Data sources:**

EMBASE, MEDLINE, NHS EED, CINAHL, EconLit, Cochrane Library, and NHS HMIC.

**Eligibility criteria for selecting studies:**

Randomised controlled trials (RCTs), cohort, case–control, before and after and cross-sectional study designs including an economic evaluation of management decisions made in any disease in secondary care within the context of an MDT meeting.

**Data extraction:**

Two independent reviewers extracted data and assessed methodological quality using the Consensus on Health Economic Criteria (CHEC-list). MDTs were defined by evidence of two characteristics: decision making requiring a minimum of two disciplines; and regular meetings to discuss diagnosis, treatment and/or patient management, occurring at a physical location or by teleconferencing. Studies that reported on the costs of administering, preparing for, and attending MDT meetings and/or the subsequent direct medical costs of care, non-medical costs, or indirect costs, and any health outcomes that were relevant to the disease being investigated were included and classified as cancer or non-cancer MDTs.

**Results:**

Fifteen studies (11 RCTs in non-cancer care, 2 cohort studies in cancer and non-cancer care, and 2 before and after studies in cancer and non cancer care) were identified, all with a high risk of bias. Twelve papers reported the frequency of meetings which varied from daily to three monthly and all reported the number of disciplines included (mean 5, range 2 to 9). The results from all studies showed mixed effects; a high degree of heterogeneity prevented a meta-analysis of findings; and none of the studies reported how the potential savings of MDT working may offset the costs of administering, preparing for, and attending MDT meetings.

**Conclusions:**

Current evidence is insufficient to determine whether MDT working is cost-effective or not in secondary care. Further studies aimed at understanding the key aspects of MDT working that lead to cost-effective cancer and non-cancer care are required.

## Introduction

Multi-disciplinary teams (MDTs) are an integrated approach to healthcare in which medical and allied health professionals consider relevant treatment options and collaboratively develop an individual treatment plan for patients [1,2]. Despite some scepticism about the effectiveness of MDT working in health care [2-4], the concept of MDT working is now widely accepted [5] and considered to be good practice in many parts of the world for the successful management of chronic disease [6-8]. Teamwork is recommended because of increasing complexity in patient management and subspecialisation of health professionals, and because of changes in working patterns in the health care sector with reduced hours of work [9].

Areas where MDT working is now commonly adopted in secondary care include care of the elderly, heart failure, stroke, mental health, and critical care. The area that has seen the most increase in MDT working, however, is in oncology where MDT working has been widely recommended including in North America [10], Australia [11,12], and continental Europe [13,14]. Indeed it is now mandated as part of the NHS Cancer Plan in the UK [6]. Studies have shown that MDT working in cancer care is associated with decreased time from diagnosis to treatment [15], more accurate pathological staging of disease [16], increased number of patients treated with radical intent [17,18], improved survival [19], and greater patient satisfaction [20]. However, these studies are largely retrospective, and there is a lack of well designed randomised controlled trials and prospective studies in this field [1,21-23]. There is also a need to consider the costs of administering, preparing for, and attending MDT meetings in any evaluation.

MDTs demand considerable organisation, management infrastructure and funding to ensure that the correct personnel are present, that the relevant patient details are collated, and that the pathological and radiological materials are reviewed by the appropriate specialists before and during the meeting [1,24,25]. Indeed, it has been shown that if critical information is absent at the MDT meeting, decisions may need to change following the meeting and this can delay the start of treatment [26]. In the UK enormous investment has been made to ensure the functioning of cancer MDT meetings and it is estimated to cost the National Health Service (NHS) around £50 million a year for preparation and a similar amount for attendance time [23]. Whether this substantial investment is justified, however, is unknown and has not been previously considered. The aim, therefore, of this paper was to systematically summarise economic studies of MDT working in secondary cancer and non-cancer care.

## Methods

### Criteria for considering studies for this review

#### Types of studies

We reviewed economic evaluations of MDT working compared to no MDT in the provision of secondary care which includes hospital-based and community-based care (Table 1). The types of study designs eligible for inclusion were randomised controlled trials (RCTs), cohort, case–control, before and after, cross-sectional, and decision analysis modelling studies.

**Table 1:** Inclusion and exclusion criteria for systematic review on cost-effectiveness of MDT working

**Inclusion criteria**	**Exclusion criteria**
Studies comparing MDT care with no MDT care	Studies that are not comparative i.e. focus on MDT care only
Secondary care services i.e. hospital-based or community-based	Primary care
Study design – RCT, cohort, case–control, before and after, cross-sectional studies, or modelling studies	Ecological studies, case reports
Applied study (i.e. studies generating primary data or modelling of secondary data)	Methodological and general articles, expert opinion, letters and abstracts
Population – persons diagnosed with any diseases	
Study setting – any country	
MDTs are defined as:	Multidisciplinary ward rounds
a) Team members from a minimum of two disciplines making decisions; and	
b) Regular team meetings to discuss diagnosis, treatment and/or patient management, occurring at a physical location or by tele-conferencing	
Outcomes - health outcomes which are relevant to the disease being investigated	
Costs – average costs of organising MDT meetings, average cost per patient treated, or incremental cost effectiveness ratios	
Journal articles, grey literature	Books
English language	Foreign languages

#### Types of participants

We included studies which enrolled people with any disease type, receiving secondary care and we classified these as cancer and non-cancer.

#### Types of interventions

MDTs were defined by evidence of both of the following characteristics:

1. Team members from a minimum of two disciplines making decisions;

2. Regular team meetings to discuss diagnosis, treatment and/or patient management, occurring at a physical location or by teleconferencing.

Where team members came from different grades within one discipline, this was considered to represent a single discipline (e.g. a consultant surgeon and a specialist surgical registrar were counted as one discipline).

Studies that evaluated MDT decision making at the patient bedside during ward rounds were excluded because we felt that they were substantially different from the type of meeting room based MDT work advocated in recent policy initiatives (e.g. for cancer services) [6,27]. The key differences between the two are that meeting room based MDTs require a coordinator, and include a systematic review of information about the patient without the patients’ input or interruptions. If a paper did not state whether the MDT meeting was ward-based or meeting room based, this was inferred from the description of the meeting.

#### Types of outcome measures

Full economic evaluations (i.e. cost-effectiveness analysis, cost-utility analysis, cost-benefit analysis and cost-consequences analysis) [28] were considered for inclusion.

#### Types of costs

We included studies that reported on the costs of supporting an MDT meeting or the subsequent direct medical costs of care, non-medical costs (e.g. patient travel), or indirect costs (e.g. productivity losses). The costs of supporting an MDT typically include the costs of team members preparing and attending MDT meetings; the latter could include travel expenses, or costs of teleconferencing, as well as administrative support provided by a team coordinator.

### Search methods for identification of studies

We searched the databases EMBASE and MEDLINE (both via the Ovid interface), NHS EED, CINAHL, EconLit, Cochrane Library, and NHS HMIC for relevant publications published from the date of inception of each database to December 2011. Grey literature was identified through the NHS HMIC database as well as based on specialised knowledge of one of the authors (JMB). The search strategy consisted of combinations of free text and MeSH terms related to the economic evaluation of an MDT (see Appendix 1). The search included journal articles and grey literature published in the English language. Reference lists from included studies and other relevant publications, including reviews, were manually checked for citations missed by the electronic search.

### Data collection and analysis

#### Selection of studies

Initially, one author (KMK) selected reports fulfilling the first inclusion criterion (see Table 1) of this review based on title and abstract. Full articles of possibly relevant studies were retrieved for more detailed evaluation. A final selection of included studies was decided by discussion with the whole team.

#### Data extraction

A data extraction template, developed using the guidelines provided by the Centre for Reviews and Dissemination [29], was used to extract the following data alongside the critical appraisal of original studies: country of investigation; objective of the study; study intervention and comparator; study design and setting; target population characteristics; sources and quality of clinical data, if applicable; sources and quality of cost data; methods for dealing with uncertainty; and study results. The following data detailing the characteristics of MDT meetings were also extracted: frequency of meetings, number of disciplines contributing, meeting venue, description of leadership style, method of dissemination of decision, and administrative support. Two authors (KMK, SS) independently extracted data. All discrepancies were reviewed and consensus achieved by discussion.

Due to the varied nature of the studies, we provide a narrative summary of the study results for our review rather than a formal meta-analysis of results.

#### Methodological quality assessment

The methodological quality of the included studies was assessed using the Consensus on Health Economic Criteria (CHEC-list), a 19-item assessment tool [30]. The CHEC-list represents a minimum set of methodological criteria that address aspects of the internal and external validity of individual economic evaluation studies. For each criterion, a study received a ‘yes’ if it had sufficiently addressed that aspect of the study, and a ‘no’ if it had not. Two authors (KMK, FEC) independently evaluated the methodological quality of included studies. Both piloted the use of the CHEC-list. Disagreement was resolved by discussion.

The six CHEC-list criteria that are regarded as most important in assessing risk of bias are appropriate choice of an time horizon, measurement of costs, valuation of costs, measurement of outcomes, valuation of outcomes, and discounting of future costs and outcomes [31]. Therefore, the risk of bias among the included studies would be high if the number of studies that fulfilled these six criteria is low.

## Results

### Overview

**Figure 1:**
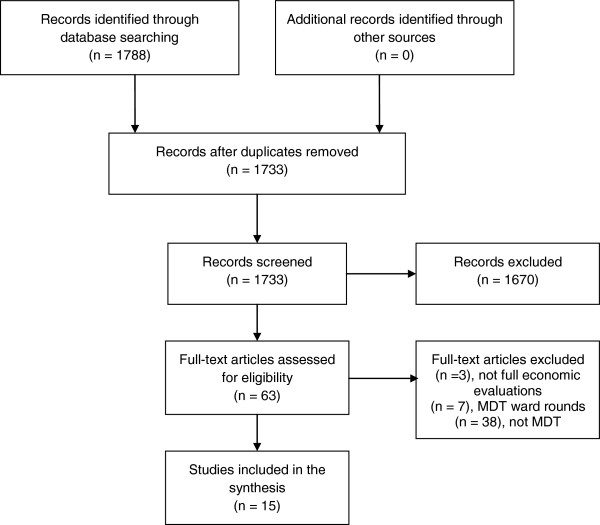
Studies selection process.

Our electronic literature search identified 1,788 articles. After a detailed review of 63 articles, 15 were included in the final analysis (Figure 1) [32-46]. Eleven were based on RCTs, [32,34,36,38-41,43-46] most commonly performed in diseases of older people [34,39,40,44] (Table 2). Only two studies evaluated MDT working in cancer care [35,37], and none were conducted in the UK. The majority of studies only included costs borne by the health care payer. No studies estimated the full cost of administering, preparing for and attending an MDT meeting.

**Table 2:** Summary of key characteristics of included studies (n = 15)

**Characteristics**	**Number of studies**
*Study country*	
USA	8
The Netherlands	2
Japan	2
Ireland	1
Italy	1
Sweden	1
*Study design*	
RCT	11
Cohort	2
Before and After	2
*Type of economic evaluation*	
Cost-consequences analysis	12
Cost-utility analysis	2
Cost-benefit analysis	1
*Disease type*	
Geriatrics	4
Cancer	2
Heart failure	2
Terminal/critical illness	2
Various	2
Mental health	1
Rheumatoid arthritis	1
Stroke	1
*Study perspective*	
Health care payer	12
Societal	3
Care setting*	
Inpatient	8
Outpatient	8
Type of decisions	
Treatment/care plan	11
Diagnosis and treatment/care plan	4

* 1 study had both inpatient and outpatient care.

### Methodological quality assessment

Only four of the nineteen criteria (i.e. research question posed in answerable form, appropriate economic study design, appropriate time horizon, and identification of important and relevant outcomes) were met by all the studies (Table 3). The choice of an appropriate time horizon is one of the six CHEC-list criteria that are regarded as important in assessing risk of bias [31]. None of the other five criteria were fulfilled by all the studies. In particular, the criteria of appropriate valuation of costs and appropriate discounting of future costs were met by very few studies – four and two respectively. The reasons for a high number of studies not meeting the criterion of appropriate valuation of costs were either a lack of information on the method of valuation, the reference year, or the use of a top-down approach in the apportionment of costs ^a^. There were four studies in the review that should have discounted future costs and outcomes because their analytic horizon was more than a year. However, only two [42,43] stated that discounting was undertaken in the analysis. Therefore, given that five of the six criteria that had an impact on the assessment of risk of bias were not met by all studies, there is a relatively high risk of bias in the results of the reviewed studies.

**Table 3:** Number of studies fulfilling each Consensus on Health Economic Criteria (CHEC-list) quality criterion

**CHEC-list quality criteria**	**Number of studies fulfilling criterion**
1. Is the study population clearly described?	12
2. Are competing alternatives clearly described?	14
3. Is a well-defined research question posed in answerable form?	15
4. Is the economic study design appropriate to the stated objective?	15
5. Is the chosen time horizon appropriate in order to include relevant costs and consequences?	15
6. Is the actual perspective chosen appropriate?	3
7. Are all important and relevant costs for each alternative identified?	0
8. Are all costs measured appropriately in physical units?	11
9. Are costs valued appropriately?	4
10. Are all important and relevant outcomes for each alternative identified?	15
11. Are all outcomes measured appropriately?	13
12. Are outcomes valued appropriately?	13
13. Is an incremental analysis of costs and outcomes of alternatives performed?	3
14. Are all future costs and outcomes discounted appropriately?	2
15. Are all important variables, whose values are uncertain, appropriately subjected to sensitivity analysis?	3
16. Do the conclusions follow from the data reported?	9
17. Does the study discuss the generalizability of the results to other settings and patient/client groups?	2
18. Does the article indicate there is no potential conflict of interest of study researcher(s) and funder(s)?	4
19. Are ethical and distributional issues discussed appropriately?	0

### Characteristics of MDT meetings

The amount of detail provided about MDT meetings varied. Twelve papers reported the frequency of meetings which varied from daily to once every three months (Table 4). The average number of disciplines in each MDT was 5 ranging from 2 to 9. Only one study stated the venue of the MDT meeting [44]. Five of the studies gave details of who led the meetings. No information was provided in any study about the amount of administrative support required for an MDT meeting. Finally, 11 reported how the MDT decisions were disseminated.

**Table 4:** Characteristics of MDT meeting

**Study**	**Frequency of meeting**	**Number of disciplines**	**Meeting venue**	**Leadership**	**Administration support**	**Dissemination of decision**
Collard et al., 1985	Twice a week	6	Not stated	Not stated	Not stated	Not stated
Williams et al., 1987	Daily	6	Outpatient clinic	Not stated	Not stated	Care plan
Timpka et al., 1997	Weekly	3	Not stated	Physicians	Not stated	Not stated
Fader et al., 1999	Biweekly	9	Not stated	Not stated	Not stated	Treatment plan
Kominski et al., 2001	At least once every 3 months	8	Not stated	Not stated	Not stated	Treatment plan
Capomolla et al., 2002	Not stated	6	Not stated	Not stated	Not stated	A report
Kasper et al., 2002	Weekly	3	Not stated	Not stated	Not stated	Treatment plan
Carling et al., 2003	Not stated	2	Not stated	Infectious disease physician	Not stated	Pro forma placed in front of patient’s chart
Van Den Hout et al., 2003	Weekly	5	Not stated	Not stated	Not stated	Not stated
Rabow et al., 2004	Regularly	8	Not stated	Team Physician	Not stated	Care plan
Yagura et al., 2005	Weekly	7	Not stated	Not stated	Not stated	Discharge plan
Gade et al., 2008	Daily	4	Not stated	Not stated	Not stated	Palliative care plan
Wolfs et al., 2009	Weekly	6	Not stated	Not stated	Not stated	Care plan
Hagiwara et al., 2011	Twice per week	4	Not stated	Hemato-oncologist	Not stated	Care plan
Pope et al., 2011	Not stated	3	Not stated	Modified Delphi method	Not stated	Not stated

### RCTs assessing cost-effectiveness of MDTs in non-cancer care

There were 11 RCTs that reported full economic evaluations of MDT care (Table 5). Four evaluated geriatric care, and the results were mixed. One RCT found that MDT directed care resulted in lower costs as well as improvement in some outcome measures. This study included two sites, and it was found that for one of the sites, total costs were lower by approximately 15%, and there were fewer complications during hospitalisation (between 3 to 12 fewer for different types of complications) [34]. However, two other RCTs of geriatric care concluded that there was no evidence of differences in costs or outcomes between MDT and no MDT care [39,44]. The fourth RCT in geriatric care showed that MDT care was associated with higher costs because the cost of implementing MDT care ^b^ for 6 months offset the reduction in medication costs, but there was no evidence of differences in functional status and survival between the two groups [40].

**Table 5:** Details of included studies (n = 15)

**Authors, date, and country**	**MDT team composition**	**Patient group or disease; sample size**	**Study design; type of economic evaluation**	**Comparison group**	**Outcome measures**	**Costs included**	**Findings**
RCTs assessing cost-effectiveness of MDTs in non-cancer care							
*Geriatric care*							
Collard et al., 1985, [34] USA	Primary nurse, social worker, physician, physical therapist, occupational therapist, medical director	Geriatric patients; treatment = 218, control = 477	RCT; cost-consequences analysis	Usual care i.e. no MDT	Health status, complications during hospitalisation, use of physical/chemical restraints at 5 months	Hospitalisation	Incremental costs of MDT – lower by US$564 (£350) per person
							Morbidity –better for MDT group
							Mortality – not measured
							Cost per QALY: not calculated
Williams et al., 1987, [44] USA	internists and family physicians with special expertise in geriatrics, psychiatrists, nurses, social workers, nutritionists	Geriatric patients; treatment = 58, controls = 59	RCT; cost-consequences analysis	Care by 1 internist	Functional status, institutional placement at 12 months	Hospitalisation, nursing home, home aid, transportation, GP visit, day centre, visits by various health professionals, meals-on-wheels, nurse & homemaker hours	Incremental costs of MDT – equivalent
							Morbidity – equivalent
							Mortality – not measured
							Cost per QALY: not calculated
Kominski et al., 2001, [39] USA	Nurses, psychiatrists, psychologists, social workers, geriatricians, nutritionists, pharmacists	Geriatric patients; treatment = 814, usual care = 873	RCT; cost-consequences analysis	Usual care i.e. no MDT	36-item Health Survey Short Form (SF-36), Mental Health Inventory (MHI) at 12 months	Inpatient, ambulatory care clinic	Incremental costs of MDT – equivalent
							Morbidity – equivalent
							Mortality – not measured
							Cost per QALY: not calculated
Pope et al., 2011, [40] Ireland	Consultant geriatrician, specialist registrar in geriatric medicine, pharmacists, nurse practitioners	Geriatric patients; treatment = 110, control = 115	RCT; cost-consequences analysis	Regular assessment i.e. no MDT	Mortality, Barthel Index, Abbreviated Mental Test Score (AMTS) at 6 months	Medical review, medication, acute hospital transfer	Incremental costs of MDT – higher by £510 per person
							Morbidity – equivalent
							Mortality – equivalent
							Cost per QALY: not calculated
*Heart failure care*							
Capomolla et al., 2002, [32] Italy	cardiologist, nurses, physiotherapists; part-time participation of dietician, psychologist, social assistant	Heart failure; treatment =112; control = 122	RCT; cost-utility analysis	Usual care by cardiologist	Death, QALY at 12 months	Pharmacologic, care management	Incremental costs of MDT – lower by US$10,768 (£6,688) per person
							Morbidity – better for the MDT group
							Mortality – lower for MDT group
							Cost per QALY: US$1,068 (£663)
Kasper et al., 2002, [38] USA	Telephone nurse co-ordinator, CHF nurse, CHF cardiologist, patient’s primary physician	heart failure; treatment = 102, control = 98	RCT; cost-consequences analysis	Care provided by GP only	Death, quality of life (QoL) at 6 months	Personnel, inpatient, outpatient pharmacy, supplies	Incremental costs of MDT – equivalent
							Morbidity – better for the MDT group
							Mortality – equivalent
							Cost per QALY: not calculated
*Terminal/critical care*							
Rabow et al., 2004, [41] USA	Social worker, nurse, chaplain, pharmacist, psychologist, art therapist, volunteer coordinator, physician	Life limiting diseases such as cancer, advanced COPD, or advanced CHF; treatment = 50, control = 40	RCT; cost-consequences analysis	Usual care i.e. no MDT	Physical functioning and symptoms, psychological, spiritual well-being at 6 months and 12 months	Office visits, emergency department visits, hospital stays	Incremental costs of MDT - equivalent
							Morbidity – better for MDT group
							Mortality – not calculated
							Cost per QALY: not calculated
Gade et al., 2008, [36] USA	Palliative care physician, nurse, hospital social worker and chaplain	A range of life-limiting diseases such as COPD, stroke, cancer; treatment = 280, control = 237	RCT; cost-consequences analysis	Usual care i.e. no MDT	Symptom severity, quality of life and survival at 6 months	Hospitalisation, pharmacologic, study	Incremental costs of MDT – lower by US$4,855 (£3,016) per patient
							Morbidity – equivalent
							Mortality – equivalent
							Cost per QALY: not calculated
Rheumatoid arthritis care, stroke care, dementia care							
Van den Hout et al., 2003, [43] The Netherlands	Nurse, rheumatologist occupational therapist, physical therapist, social worker.	Rheumatoid arthritis; treatment = 71 (inpatient MDT), 68 (outpatient MDT) control = 71	RCT; cost-consequences analysis	Usual care i.e. no MDT	Functional status, quality of life at 6, 12, 26, 52, and 104 weeks	Hospitalisations, personnel, home nursing care, other health professionals drugs, and appliances, out of pocket, home care informal care, paid and unpaid labour	Incremental costs of MDT –higher by €5,160 to €10,876 (£4,230 to £8,915) per person
							Morbidity – equivalent
							Mortality – not measured
							Cost per QALY: not calculated
Yagura et al., 2005, [46] Japan	Physicians, nurses, physical therapists, occupational therapists, speech therapists, clinical psychologists, social worker	Stroke; treatment = 91, control = 87	RCT; cost-consequences analysis	Usual care i.e. no MDT	Functional status, impairment status (duration of measurement not stated)	Hospitalisation	Incremental costs of MDT –equivalent
							Morbidity – equivalent
							Mortality – not measured
							Cost per QALY: not calculated
Wolfs et al., 2009, [45] The Netherlands	Old age psychiatry, geriatric medicine, neuropsychology, physiotherapy, occupational therapy, geriatric nursing and mental health nursing	Patients suspected of having dementia or a cognitive disorder; treatment = 131, control = 88	RCT; cost-utility analysis	Usual care i.e. no MDT	QALYs, cognition and behavioural problems at 6 months and 12 months	Medical, informal care, out-of-pocket	Incremental costs of MDT – higher by €65 (£53) per person
							Morbidity – better for MDT group
							Mortality – not measured
							Cost per QALY: €1,267 (£1,039)
Other study designs assessing cost-effectiveness of MDTs in non-cancer care							
Timpka et al., 1997, [42] Sweden	Part-time physicians, psychologist, social workers	Patients with chronic minor diseases and long-term absence from working life; 239	Cohort; cost-benefit analysis	baseline characteristics before start of programme	Vocational activity, benefits to society at 12 months and 5 years	Programme, indirect	Costs – 30,000 SEK (£2,852) per person
							Benefits – 1.25 million SEK (£117,500) per person
							Cost-benefit ratio – 4.9
Carling et al., 2003, [33] USA	Clinical pharmacist, infectious diseases physician	Adults receiving parenteral 3rd generation cephalosporins, aztreonam, parenteral fluoroquinolones, or imipenem; sample size not specified	B&A; cost-consequences analysis	Before MDT implemented	Incidence of nosocomial infections per 1000 patient-days	Medication	Incremental costs of MDT – lower by US$200,000 to US$250,000 per year (£124,224 to £155,280)
							Morbidity – better for MDT group
							Mortality – not measured
							Cost per QALY: not calculated
Studies assessing cost-effectiveness of MDT in cancer care							
Fader et al., 1998, [35] USA	specialists in dermatology; surgical, medical, & radiation oncology; plastic & dermatologic surgery; otorhinolaryngology; obstetrics/gynecology; ophthalmology, dermatopathology; nuclear medicine; and social work	Melanoma; treatment = 104, control = 104	Cohort; cost-consequences analysis	Usual care i.e. no MDT	Surgical morbidity at 1 month, survival at 5 years	Diagnosis and initial management	Incremental costs of MDT – lower by US$1,595 (£991) per person
							Morbidity – equivalent
							Mortality – equivalent
							Cost per QALY: not calculated
Hagiwara et al., 2011, [37] Japan	Hemato-oncologist, nurse, dietitian, pharmacist	Hematologic malignancies; Before – 67, After – 102	B&A; cost-consequences analysis	No MDT	Number of adverse events, death (duration of measurement not stated)	Parenteral nutrition, antibiotics, food and nutritional supplement, MDT personnel	Incremental costs of MDT – lower by 403,600 yen (£3,058) per person
							Morbidity – better for MDT group
							Mortality – equivalent
							Cost per QALY: not calculated

*B&A *before and after, *CHF *chronic heart failure, *RCT* randomised controlled trial.

Similarly, there were inconsistent results from two RCTs of MDT care for patients suffering from heart failure. One of them found that MDT care was cost-effective with an incremental cost-effectiveness ratio of US$1,068 per Quality Adjusted Life Year (QALY) (£663/QALY, £1 = US$1.61, rate as at 20 April 2012) [32]. However, the other RCT showed that there was no evidence of a real cost difference but quality of life improved when comparing MDT and no-MDT care [38].

Two RCTs evaluated MDT care amongst patients with terminal conditions; the results were mixed. One reported that costs were lower for the MDT group by about 30%, but there was no evidence of any differences in measures of symptom control, survival, and quality of life between the MDT and no MDT groups [36]. In contrast, the other study showed that there was no evidence of a real cost difference but some outcome measures such as dyspnea, anxiety, spiritual well-being, improved when comparing MDT and no-MDT care [41].

The last three RCTs, each of people with a different disease, also showed varied results. One study relating to patients with mental health problems found that MDT care was cost-effective with an incremental cost effectiveness ratio of €1,267/QALY (£1,039/QALY, £1 = €1.22, rate as at 20 April 2012) [45]. However, a RCT concerning stroke patients concluded that there was no evidence of real cost or outcome measures differences [46]. Furthermore, in a study that investigated the impact of MDT care versus no MDT care among patients diagnosed with rheumatoid arthritis, there was no evidence of differences in outcome measures but costs were higher in the MDT care group by approximately 30% to 50% [43].

### Other study designs assessing cost-effectiveness of MDTs in non-cancer care

A prospective cohort study based in Sweden which considered the effect of MDT on vocational activity among patients with chronic minor diseases and long-term absence from working life, reported a total discounted cost of 7.2 million SEK (£684,410, £1 = 10.52 SEK, rate as at 20 April 2012), total benefits of 35.1 million SEK (£3.3 million), and hence, a positive cost-benefit ratio of 4.9 [42]. No estimate for cost per person was provided. In addition, a ‘before and after’ study found that the rate of infection per 1000 patient days after the implementation of MDT antibiotic management care fell by between 25% to 70% and costs decreased by about 30% [33].

### Studies assessing cost-effectiveness of MDTs in cancer care

There were two non-randomised studies that reported results of MDTs in cancer care (Table 5). In a retrospective cohort study of MDT versus no MDT care for patients with malignant melanoma, the costs of health care were 33% to 50% lower in patients whose management decisions were made by an MDT [35]. However, there was no evidence of any differences in wound complication and 5-year survival rates. Hagiwara et al. (2011) [37] reported clinical outcomes before and after the introduction of a multidisciplinary nutritional team for patients with haematologic malignancies. It was found that incidences of hepatic complications, hyperglycemia, and central venous catheter infection were lower in the ‘after’ group than in the ‘before’ group, and costs fell by about 20%.

## Discussion

### Summary of findings

We identified fifteen primary studies on the cost-effectiveness of MDT decision making in secondary care, of which only two concerned cancer and the others covered a wide range of disease types. The characteristics of MDT meetings were diverse. The existing evidence was limited and there was a relatively high risk of bias amongst the studies. Whilst both studies relating to cancer care showed that MDTs were associated with lower costs and better outcomes or outcomes comparable to no MDT care, neither study was a RCT. The results from eleven RCTs assessing the cost-effectiveness of MDTs in non-cancer care were very varied. Only three of these showed that MDT working in secondary care had a positive effect on outcomes and costs. However, the degree of this positive impact varied. Moreover, the positive impact of MDT on some costs components might be offset by the full cost of administering, preparing for and attending an MDT meeting, for which no estimates were reported in the included studies. Therefore, based on existing evidence, it is not possible to determine if MDT working is cost-effective or not in secondary cancer and non-cancer care.

### Strengths and weaknesses of this systematic review

To our knowledge, we conducted the first systematic review on the cost-effectiveness of MDT working in secondary care, and we explicitly attempted to capture all types of costs, especially the significant costs of supporting MDT working. The limitations of this review need to be borne in mind. Firstly, as with any systematic review, publication bias might be a problem if studies with null findings have not been published. Secondly, this review was not restricted to a particular country or disease type. While this approach is valuable in describing the available evidence from a wide perspective, it restricts the comparability across studies. Given that the studies were diverse, particularly in the type of care offered in the comparison groups, the findings may not be applicable across different healthcare settings where different clinical practices and geographical constraints operate. The operationalising of MDT may also differ in different settings and together with the variability in reporting, there may be studies that have been conceived as MDT, but based on our interpretation, were not. However, we tried to be inclusive in our definition of MDT working so that we did not exclude potentially relevant studies.

### Weaknesses of the evidence

We have identified four key weaknesses in the existing literature on the cost-effectiveness of MDT working. First, there was no standard definition or operationalisation of MDT working evident in the literature. Most studies did not provide key details of the MDT meeting venue, leadership (i.e. who leads the meeting and or how are decisions made), or administrative support. Without a common definition or clear description of MDTs, it was difficult to compare results between studies or draw conclusions on the cost-effectiveness of MDT decision making. Second, the full costs of administering, preparing for and attending an MDT meeting were not estimated in the included studies. Given that the MDT meeting is a key feature of MDT working, the omission of the associated costs in any cost-effectiveness analysis of MDT could lead to an underestimation of costs, and hence bias in the results. Third, the methodological quality assessment has demonstrated that there was a relatively high risk of bias amongst the studies. This meant that the reliability and reproducibility of the included studies were compromised, and the ability to determine conclusions from our systematic review was restricted. Fourth, the available evidence was limited so that it was not possible to draw firm conclusions as to whether MDT working in cancer or non-cancer care would lead to cost-effective care or not.

### Comparison with other studies

In their review to determine the critical elements of effective team-working in patient care teams, Bosch et al. [47] concluded that current literature provided little insight into the underlying mechanisms. They reported results relating to costs and resource utilisation but not on cost-effectiveness of MDTs. In comparison, our systematic review focused specifically on full economic evaluations of MDT working, and sought to include studies that estimated the costs of supporting MDT working. In another review examining the association between cancer MDTs and survival, the authors also highlighted the lack of a consistent definition of MDT [21]. This directly hampers any investigation of its effectiveness and costs. While none of the included studies in this review had accounted for the full costs of administering, preparing for and attending an MDT meeting, an audit conducted at a large teaching hospital/cancer centre estimated the average annual cost of MDTs in salaries alone was about £1 million [48]. Time spent preparing for MDT meetings could be considerable. A study examining the participation of pathologists and radiologists in MDT meetings found that the former spent about an average of 2.4 hours, and the latter 2 hours in preparation [49]. Although our review did not find evidence that MDT working is cost-effective in cancer care, there appears to be a growing consensus that telemedicine delivered MDT cancer meetings are more cost-effective than ‘in-person’ meetings [50-53].

### Future research

Based on the findings of our systematic review, we propose that three issues need to be addressed in future research in this area. Firstly, future research should ensure a clear definition of MDT working and detailed reporting of its characteristics. In our systematic review, we found that the description of the two key features of MDT working was so vague that it was difficult to determine whether MDT working had taken place or not. We recommend that studies should provide the characteristics of the MDT meeting as shown in Table 4. Secondly, it is essential to estimate the full costs of administering, preparing for and attending an MDT meeting. Such attempts should take into account two issues. One, all relevant costs should be identified. We recommend that the following costs should be accounted for: time spent by each team member in preparing for the MDT meeting and this should include time spent by team coordinator in providing administrative support to the MDT meeting, time spent on travelling to and from meetings by any team member who is located on a different site than the meeting venue, costs of using tele-conferencing if applicable, and time spent by each member at each meeting. Two, these costs should be measured as accurately as possible. The standard methods used in health services research for workflow assessments are work sampling, time efficiency, and time-and-motion. The first two methods are less resource intensive as compared to the third method but there is evidence to show that the time-and-motion technique may produce the most accurate results [54-56]. Thirdly, it remains unclear what models of MDT are cost-effective in secondary care. Therefore, future studies using strong study designs should compare the cost-effectiveness of different models or aspects of MDT working ^c^. For this to be performed accurately there is a need for agreement in the clinical community about which outcomes would be core in the evaluation of MDT working. Whilst some clinical outcomes are important, the inclusion of patient-centred outcomes and satisfaction of health professionals as well as assessment of the quality of decision-making and MDT decision-implementation may also be important [2,57,58].

### Policy implications

Without further research that addresses the three key issues highlighted in the previous section, it would be hard to make firm policy recommendations. The cost-effectiveness of MDT working in secondary care is likely to vary according to many factors, such as cost of supporting MDT working, disease type, treatment selected, and geographical location. The costs of supporting MDT working are directly linked to factors that affect management decisions of MDTs, such as inclusion of time to prepare for MDTs into team-members’ job plans, making team and leadership skills training available to team-members, and systematic input from nursing personnel [59]. The commitment of resources to these factors will differ across settings. Therefore, it is likely that there is no ‘one-size-fits-all’ model of MDT working to suit all diseases, treatment modalities and locations.

## Conclusion

The widespread adoption of MDT working in cancer and non-cancer care has occurred despite a lack of evidence on cost-effectiveness. The available evidence is limited and at relatively high risk of bias. It is not possible to determine if MDT working in cancer and non-cancer care is cost-effective or not. Further studies aimed at understanding the key aspects of MDT working that lead to cost-effective care, and the costs of supporting MDT working are required.

## Endnotes

^a^ Costs can be derived by a bottom-up or top-down approach. In a bottom-up approach, costs are determined by examining individual utilisation. In a top-down approach, costs are apportioned across individuals, and this may be less accurate due to questionable assumptions used in the apportionment of costs.

^b^ Personal communication with the lead author has confirmed that these costs did not include the full costs of administering, preparing for and attending an MDT meeting.

^c^ As MDT working is now so prevalent in secondary care, especially cancer care, it might not be possible to compare the cost-effectiveness of no-MDT versus MDT working.

## Appendix 1

Search Strategy

EMBASE

#1. multidisciplinary OR “multidisciplinary team” OR interdisciplinary OR “interdisciplinary team” OR team* OR “patient care team” OR “health care team*” OR “medical care team”

#2. “cost savings” OR “cost-effective*” OR “cost-benefit” OR “cost-utility” OR “economic evaluation” OR economic* OR “cost analysis” OR “health care costs”

#3. hospital or “hospital setting” OR “hospital based” OR “secondary care”

#4. #1 AND #2 AND #3

#5. limit to (human and English language) AND (article)

MEDLINE (MeSH terms used)

#1. (Patient Care Team)

#2. ((Costs and Cost Analysis) OR (Economic*) OR (Cost-Benefit Analysis) OR (Cost Savings) OR (Health Care Costs) OR (Cost of Illness) OR (Health Services Accessibility) OR (Patient Transfer) OR (Travel) OR (Transportation of Patients)

#3. #1 AND #2

#4. limit #3 to (English language and humans)

NHS EED

(multidisciplinary OR “multidisciplinary team*” OR interdisciplinary OR “interdisciplinary health team*” OR team* OR “patient care team*” OR “health care team*” OR “medical care team*”) (english:la)

CINAHL

#1. multidisciplinary OR “multidisciplinary team*” OR interdisciplinary OR “interdisciplinary health team*” OR team* OR “patient care team*” OR “health care team*” OR “medical care team*”

#2. “cost savings” OR “cost-effective*” OR “cost-benefit” OR “cost-utility” OR “economic evaluation” OR economic* OR “cost analysis” OR ‘health care costs’ OR access* OR travel OR burden

#3. #1 AND #2

#4. limit to English Language; Peer Reviewed; Research Article; Exclude MEDLINE records.

EconLit

#1. multidisciplinary OR “multidisciplinary team*” OR interdisciplinary OR “interdisciplinary health team*” OR team* OR “patient care team*” OR “health care team*” OR “medical care team*”

#2. “cost savings” OR “cost-effective*” OR “cost-benefit” OR “cost-utility” OR “economic evaluation” OR economic* OR “cost analysis” OR ‘health care costs’ OR access* OR travel OR burden

#3. #1 AND #2

#4 limit to (English language) AND (article or conference paper)

Cochrane Library

multidisciplinary OR “multidisciplinary team*” OR interdisciplinary OR “interdisciplinary health team*” OR team* OR “patient care team*” OR “health care team*” OR “medical care team*” in Title, Abstract or Keyword and “cost savings” OR “cost-effective*” OR “cost-benefit” OR “cost-utility” OR “economic evaluation” OR economic* OR “cost analysis” OR ‘health care costs’ OR access* OR travel OR burden in in Title, Abstract or Keyword

NHS HMIC

(multidisciplinary OR “multidisciplinary team*” OR interdisciplinary OR “interdisciplinary health team*” OR team* OR “patient care team*” OR “health care team*” OR “medical care team*”) (english:la)

## Competing interests

The authors declare that they have no competing interests.

## Authors’ contributions

KMK, JMB, ARN, WH conceived of the study and participated in its design. All authors analysed the data, drafted the manuscript, and read and approved the final manuscript.

## References

[B1] Fleissig A Multidisciplinary teams in cancer care: are they effective in the UK? Lancet Oncol 2006 7 11 935 943 10.1016/S1470-2045(06)70940-8 17081919

[B2] Molleman E Consequences of participating in multidisciplinary medical team meetings for surgical, nonsurgical, and supporting specialties Med Care Res Rev 2010 67 2 173 193 10.1177/1077558709347379 19815682

[B3] Lemieux-Charles L McGuire WL What do we know about health care team effectiveness? A review of the literature Med Care Res Rev 2006 63 3 263 300 10.1177/1077558706287003 16651394

[B4] Rodriguez HP The effect of care team composition on the quality of HIV care Med Care Res Rev 2008 65 1 88 113 18184871 10.1177/1077558707310258

[B5] Baker DP Day R Salas E Teamwork as an essential component of high-reliability organizations Health Serv Res 2006 41 4 Pt 2 1576 1598 16898980 10.1111/j.1475-6773.2006.00566.x PMC1955345

[B6] Department of Health The NHS cancer plan: a plan for investment, a plan for reform 2000 London: Department of Health

[B7] Department of Health The NHS plan: a plan for investment, a plan for reform 2000 London: Department of Health

[B8] Institute of Medicine Crossing the quality chasm: a new health system for the 21st century 2001 Washington D.C: National Academies Press 25057539

[B9] Wagner EH The role of patient care teams in chronic disease management BMJ 2000 320 7234 569 572 10.1136/bmj.320.7234.569 10688568 PMC1117605

[B10] Tripathy D Multidisciplinary care for breast cancer: barriers and solutions Breast J 2003 9 1 60 63 10.1046/j.1524-4741.2003.09118.x 12558678

[B11] Luxford K Rainbird K Multidisciplinary care for women with breast cancer: a national demonstration program N S W Public Health Bull 2001 12 10 277 279 10.1071/NB01093 12105437

[B12] Zorbas H Multidisciplinary care for women with early breast cancer in the Australian context: what does it mean? Med J Aust 2003 179 10 528 531 14609416 10.5694/j.1326-5377.2003.tb05678.x

[B13] Magnani T The 6-year attendance of a multidisciplinary prostate cancer clinic in Italy: incidence of management changes BJU Int 2012 110 7 998 1003 10.1111/j.1464-410X.2012.10970.x 22404874

[B14] van Nes J an de Velde C The multidisciplinary breast cancer team: promoting better care Ned Tijdschr Geneeskd 2005 149 1929 1931 16159029

[B15] Gabel M Hilton NE Nathanson SD Multidisciplinary breast cancer clinics. Do they work? Cancer 1997 79 12 2380 2384 10.1002/(SICI)1097-0142(19970615)79:12<2380::AID-CNCR12>3.0.CO;2-N 9191526

[B16] Davies AR The multidisciplinary team meeting improves staging accuracy and treatment selection for gastro-esophageal cancer Dis Esophagus 2006 19 6 496 503 10.1111/j.1442-2050.2006.00629.x 17069595

[B17] Junor EJ Hole DJ Gillis CR Management of ovarian cancer: referral to a multidisciplinary team matters Br J Cancer 1994 70 2 363 370 10.1038/bjc.1994.307 8054286 PMC2033481

[B18] Kingsmore D Hole D Gillis C Why does specialist treatment of breast cancer improve survival? The role of surgical management Br J Cancer 2004 90 10 1920 1925 10.1038/sj.bjc.6601846 15138472 PMC2409479

[B19] Kesson EM Effects of multidisciplinary team working on breast cancer survival: retrospective, comparative, interventional cohort study of 13 722 women BMJ 2012 344 e2718 10.1136/bmj.e2718 22539013 PMC3339875

[B20] Boxer MM Do multidisciplinary team meetings make a difference in the management of lung cancer? Cancer 2011 117 22 5112 5120 10.1002/cncr.26149 21523766

[B21] Hong NJ Examining the potential relationship between multidisciplinary cancer care and patient survival: an international literature review J Surg Oncol 2010 102 2 125 134 10.1002/jso.21589 20648582

[B22] Tattersall MH Multidisciplinary team meetings: where is the value? Lancet Oncol 2006 7 11 886 888 10.1016/S1470-2045(06)70916-0 17081913

[B23] Taylor C Multidisciplinary team working in cancer: what is the evidence? BMJ 2010 340 c951 10.1136/bmj.c951 20332315

[B24] Department of Health Guidance on commissioning cancer services: Improving outcomes in upper gastro-intestinal cancers 2001 London: Department of Health

[B25] McNair AG Maximising recruitment into randomised controlled trials: the role of multidisciplinary cancer teams Eur J Cancer 2008 44 17 2623 2626 10.1016/j.ejca.2008.08.009 18804999

[B26] Goolam-Hossen T Waiting times for cancer treatment: the impact of multi-disciplinary team meetings Behaviour & Information Technology 2011 30 4 467 471 10.1080/0144929X.2011.55374723570067

[B27] NSW Department of Health Multidisciplinary ward rounds: A resource 2011 Sydney: NSW Department of Health

[B28] Drummond MF Methods for the economic evaluation of health care programmes 2005 3 Oxford: Oxford University Press

[B29] Centre for Reviews and Dissemination Systematic reviews: CRD’s guidance for undertaking reviews in health care 2009 York: CRD, University of York

[B30] Evers S Criteria list for assessment of methodological quality of economic evaluations: Consensus on Health Economic Criteria Int J Technol Assess Health Care 2005 21 2 240 245 15921065

[B31] Uegaki K Economic evaluations of occupational health interventions from a corporate perspective - a systematic review of methodological quality Scand J Work Environ Health 2010 36 4 273 288 10.5271/sjweh.3017 20473477

[B32] Capomolla S Cost/utility ratio in chronic heart failure: comparison between heart failure management program delivered by day-hospital and usual care J Am Coll Cardiol 2002 40 7 1259 1266 10.1016/S0735-1097(02)02140-X 12383573

[B33] Carling P Favorable impact of a multidisciplinary antibiotic management program conducted during 7 years Infect Control Hosp Epidemiol 2003 24 9 699 706 10.1086/502278 14510254

[B34] Collard AF Bachman SS Beatrice DF Acute care delivery for the geriatric patient: an innovative approach QRB Qual Rev Bull 1985 11 6 180 185 3939598

[B35] Fader DJ The multidisciplinary melanoma clinic: a cost outcomes analysis of specialty care J Am Acad Dermatol 1998 38 5 Pt 1 742 751 9591819 10.1016/s0190-9622(98)70203-8

[B36] Gade G Impact of an inpatient palliative care team: a randomized control trial J Palliat Med 2008 11 2 180 190 10.1089/jpm.2007.0055 18333732

[B37] Hagiwara S Multidisciplinary nutritional support for autologous hematopoietic stem cell transplantation: a cost-benefit analysis Nutrition 2011 27 11–12 1112 1117 21482071 10.1016/j.nut.2010.11.010

[B38] Kasper EK A randomized trial of the efficacy of multidisciplinary care in heart failure outpatients at high risk of hospital readmission J Am Coll Cardiol 2002 39 3 471 480 10.1016/S0735-1097(01)01761-2 11823086

[B39] Kominski G UPBEAT: the impact of a psychogeriatric intervention in VA medical centers. Unified Psychogeriatric Biopsychosocial Evaluation and Treatment Med Care 2001 39 5 500 512 10.1097/00005650-200105000-00010 11317098

[B40] Pope G Specialist medication review does not benefit short-term outcomes and net costs in continuing-care patients Age Ageing 2011 40 3 307 312 10.1093/ageing/afq095 20817937

[B41] Rabow MW The comprehensive care team: a controlled trial of outpatient palliative medicine consultation Arch Intern Med 2004 164 1 83 91 10.1001/archinte.164.1.83 14718327

[B42] Timpka T Long-term economic effects of team-based clinical case management of patients with chronic minor disease and long-term absence from working life Scand J Soc Med 1997 25 4 229 237 9460135 10.1177/140349489702500402

[B43] van den Hout WB Cost effectiveness and cost utility analysis of multidisciplinary care in patients with rheumatoid arthritis: a randomised comparison of clinical nurse specialist care, inpatient team care, and day patient team care Ann Rheum Dis 2003 62 4 308 315 10.1136/ard.62.4.308 12634227 PMC1754484

[B44] Williams ME How does the team approach to outpatient geriatric evaluation compare with traditional care: a report of a randomized controlled trial J Am Geriatr Soc 1987 35 12 1071 1078 3119693 10.1111/j.1532-5415.1987.tb04923.x

[B45] Wolfs CA Economic evaluation of an integrated diagnostic approach for psychogeriatric patients: results of a randomized controlled trial Arch Gen Psychiatry 2009 66 3 313 323 10.1001/archgenpsychiatry.2008.544 19255381

[B46] Yagura H Patients with severe stroke benefit most by interdisciplinary rehabilitation team approach Cerebrovasc Dis 2005 20 4 258 263 10.1159/000087708 16123546

[B47] Bosch M Effectiveness of patient care teams and the role of clinical expertise and coordination: a literature review Med Care Res Rev 2009 66 6 Suppl 5S 35S 10.1177/1077558709343295 19692553

[B48] Fosker CJ Dodwell D The cost of the MDT [Rapid Response] 2010 http://www.bmj.com/rapid-response/2011/11/02/cost-mdt. accessed on 23 August 2012

[B49] Kane B Multidisciplinary team meetings and their impact on workflow in radiology and pathology departments BMC Med 2007 5 15 10.1186/1741-7015-5-15 17567904 PMC1919390

[B50] Kitamura C Zurawel-Balaura L Wong RK How effective is video consultation in clinical oncology? A systematic review Curr Oncol 2010 17 3 17 27 20567623 10.3747/co.v17i3.513 PMC2880899

[B51] Kunkler IH TELEMAM: a cluster randomised trial to assess the use of telemedicine in multi-disciplinary breast cancer decision making Eur J Cancer 2007 43 17 2506 2514 10.1016/j.ejca.2007.08.026 17962011

[B52] Stalfors J Bjorholt I Westin T A cost analysis of participation via personal attendance versus telemedicine at a head and neck oncology multidisciplinary team meeting J Telemed Telecare 2005 11 4 205 210 10.1258/1357633054068892 16007751

[B53] Westin T Stalfors J Tumour boards/multidisciplinary head and neck cancer meetings: are they of value to patients, treating staff or a political additional drain on healthcare resources? Curr Opin Otolaryngol Head Neck Surg 2008 16 2 103 107 10.1097/MOO.0b013e3282f6a4c4 18327027

[B54] Ampt A A comparison of self-reported and observational work sampling techniques for measuring time in nursing tasks J Health Serv Res Policy 2007 12 1 18 24 10.1258/135581907779497576 17244393

[B55] Burke TA A comparison of time-and-motion and self-reporting methods of work measurement J Nurs Adm 2000 30 3 118 125 10.1097/00005110-200003000-00003 10725940

[B56] Finkler SA A comparison of work-sampling and time-and-motion techniques for studies in health services research Health Serv Res 1993 28 5 577 597 8270422 PMC1069965

[B57] Blazeby JM Analysis of clinical decision-making in multi-disciplinary cancer teams Ann Oncol 2006 17 3 457 460 10.1093/annonc/mdj102 16322114

[B58] Nallamothu BK Cohen DJ No "i" in Heart Team: incentivizing multidisciplinary care in cardiovascular medicine Circ Cardiovasc Qual Outcomes 2012 5 3 410 413 10.1161/CIRCOUTCOMES.112.966101 22592755

[B59] Lamb BW Quality of care management decisions by multidisciplinary cancer teams: a systematic review Ann Surg Oncol 2011 18 8 2116 2125 10.1245/s10434-011-1675-6 21442345

